# Etiologic characteristics revealed by mNGS-mediated ultra-early and early microbiological identification in airway secretions from lung transplant recipients

**DOI:** 10.3389/fimmu.2023.1271919

**Published:** 2023-09-21

**Authors:** Xiaoqin Zhang, Xuemei Tang, Xiaoli Yi, Yu Lei, Sen Lu, Tianlong Li, Ruiming Yue, Lingai Pan, Gang Feng, Xiaobo Huang, Yiping Wang, Deyun Cheng

**Affiliations:** ^1^ Department of Respiratory and Critical Care Medicine, West China Hospital, West China Clinical Medical School, Sichuan University, Chengdu, China; ^2^ Department of Critical Care Medicine, Sichuan Provincial People’s Hospital, University of Electronic Science and Technology of China, Chengdu, China; ^3^ Medical Department, Genoxor Medical Science and Technology Inc., Shanghai, China; ^4^ Department of Thoracic Surgery, Sichuan Provincial People’s Hospital, University of Electronic Science and Technology of China, Chengdu, China

**Keywords:** lung transplant, mNGS, airway secretions, early infection, etiology

## Abstract

**Background:**

Post-operative etiological studies are critical for infection prevention in lung transplant recipients within the first year. In this study, mNGS combined with microbial culture was applied to reveal the etiological characteristics within one week (ultra-early) and one month (early) in lung transplant recipients, and the epidemiology of infection occurred within one month.

**Methods:**

In 38 lung transplant recipients, deep airway secretions were collected through bronchofiberscope within two hours after the operation and were subjected to microbial identification by mNGS and microbial culture. The etiologic characteristics of lung transplant recipients were explored. Within one month, the infection status of recipients was monitored. The microbial species detected by mNGS were compared with the etiological agents causing infection within one month.

**Results:**

The detection rate of mNGS in the 38 airway secretions specimens was significantly higher than that of the microbial culture (P<0.0001). MNGS identified 143 kinds of pathogenic microorganisms; bacterial pathogens account for more than half (72.73%), with gram-positive and -negative bacteria occupying large proportions. Fungi such as *Candida* are also frequently detected. 5 (50%) microbial species identified by microbial culture had multiple drug resistance (MDR). Within one month, 26 (68.42%) recipients got infected (with a median time of 9 days), among which 10 (38.46%) cases were infected within one week. In the infected recipients, causative agents were detected in advance by mNGS in 9 (34.62%) cases, and most of them (6, 66.67%) were infected within one week (ultra-early). In the infection that occurred after one week, the consistency between mNGS results and the etiological agents was decreased.

**Conclusion:**

Based on the mNGS-reported pathogens in airway secretions samples collected within two hours, the initial empirical anti-infection regimes covering the bacteria and fungi are reasonable. The existence of bacteria with MDR forecasts the high risk of infection within 48 hours after transplant, reminding us of the necessity to adjust the antimicrobial strategy. The predictive role of mNGS performed within two hours in etiological agents is time-limited, suggesting continuous pathogenic identification is needed after lung transplant.

## Introduction

Although the first human lung transplant was performed in 1963, the operation became a clinical reality for treating end-stage lung diseases until the mid-1980s, after overcoming most surgical and pharmacologic challenges (1, 2). Nevertheless, the morbidity and mortality remain high, and the survival rate in lung transplant recipients is lower than of other solid organ transplant recipients, with a 5-year survival rate of 55.6% (3). Rejection- and infection-related complications are the main factors for overall morbidity and mortality in lung transplant recipients (4, 5). For lung transplant recipients, infection is a significant complication. It represents the most common cause of death within the first year, and pulmonary infection-related respiratory failure is the leading cause of death during post-operative admission (<30 days) (6, 7). So far, most of the post-operative etiological studies in lung transplants mainly focus on the episodes of infection that occurred within three months or one year following the operation. In a previous epidemiological study in which 51 lung transplant recipients were followed for a mean of 38.2 months, 42% of infectious episodes occurred within the first three months, and 75% developed within the first year after transplant (8). However, infections that occur within one week (ultra-early) and one month (early) after transplantation are rarely paid attention to, and the associated etiological study is insufficient.

Traditional etiological diagnosis methods of bronchoscopy specimens include airway secretions for microbial culture, smear microscopy, and histopathology (9). In lung transplant recipients, airway secretions microbial culture is the most frequently adopted for etiological examination to diagnose pulmonary infection (10, 11). However, the positive rate of microbial culture is low because of the limitation in microbial cultivating techniques and the impact of lesions surrounded by fibrous tissue and antibiotic application history (9). Consequently, molecular diagnostic technologies are emerging as complementary methodologies for pathogenic detection (12), including the polymerase chain reaction that focuses on a specific pathogen (13).

Metagenomic next-generation sequencing (mNGS), an unbiased and practical approach for pathogen identification with a shorter turn-around time, has been employed to diagnose infectious diseases (14). In liver transplant recipients, mNGS was adopted in the diagnosis and treatment guidance of post-operative infection, showing distinct advantages in detecting mixed, viral, and parasitic infections over the traditional culture method (15). Compared with urine culture, mNGS performed more remarkably in etiological diagnosis for kidney transplant recipients with urinary tract infections (16). In lung transplant recipients, mNGS is committed to pathogenic detection in airway secretions samples, with a shorter turn-around time, providing timely information for diagnosing pulmonary infections (17). These findings highlight the great potential of mNGS in detecting pathogenic microorganisms and identifying infection in lung transplant recipients. Herein, the secretions samples were absorbed through a bronchofiberscope from the deep airway within two hours after lung transplant. Airway secretions were subjected to mNGS test and microbial culture to reveal the ultra-early microbial characteristics and analyze the pulmonary infection within one month in recipients. Our data may offer a critical reference for antimicrobial regimens to prevent infections developed within one week or month, thereby reducing the related mortality.

## Materials and methods

### Lung transplant recipient enrollment

Patients undergoing lung transplantation at Sichuan Provincial People’s Hospital from October 2018 to June 2022 were included in this study. The inclusive and exclusive criteria for donor lungs were described in our previous study (18), and listed as follows.

Donor lungs inclusion criteria: (a) Age < 60 years old, smoking history < 20 packs/year. (b) No chest injury. (c) Continuous mechanical ventilation < 1 week. (d) PaO_2_ > 300 mmHg (FiO_2_ = 100%, PEEP = 5cm H_2_O). (e) X-ray or CT shows that the lung field is relatively clear. (f) No abscess secretion was found through bronchoscopy in the lung bronchus.

Donor lungs exclusion criteria: (a) Age > 60 years old, smoking history > 20 packs/year. (b) Chest trauma and lung contusion. (c) Continuous mechanical ventilation > 1 week. (d) PaO_2_ < 300 mmHg (FiO_2_ = 100%, PEEP = 5cm H_2_O). (e) X-ray or CT shows that the lung field is infected. (f) There are purulent secretions at bronchoscopy in the donor’s lower airways. (g) The percentage of white blood cells, neutrophils, C-reactive protein, and procalcitonin increases gradually compared with the situation at the onset of the disease. (h) The donor’s body temperature is higher than normal. (i) Blood culture is positive.

### Study design and sample collection

Basic information about the enrolled recipients, including age, sex, primary indications for a lung transplant, types of lung transplantation (bilateral or unilateral), and infection status within one month following the operation, was recorded. Prognostic information on the enrolled patients’ antimicrobial use, mechanical ventilation, and ICU hospitalization was recorded in detail.

In most lung transplant centers in China, timely bronchofiberscopy after surgery is a routine examination aiming to clean the airway secretions through a bronchofiberscope, which helps to avoid obstructing the small airway and reduce pathogens. Therefore, airway secretions were absorbed from the deep airway by bronchofiberscope two hours after the operation and sent for traditional microbial culture and mNGS for pathogen detection immediately. In the following days, within one month, airway secretions or BALFs were collected for microbial culture every few days, depending on the actual conditions in recipients. Microbial culture for the above samples was conducted in our hospital. The yielded pathogen spectrum was analyzed and compared between these two methods. The incidence of infection within one month and the occurrence time in these recipients were determined. The causative agents for infection were compared with the pathogenic microorganisms reported by mNGS in airway secretions collected within two hours to evaluate the role of mNGS in forewarning potential pathogens.

### mNGS procedure

The whole process of mNGS was completed by Genoxor Medical Science and Technology Inc. (Shanghai, China). The airway secretions samples were stored at 4°C and sent for mNGS detection within 24h. These steps included pre-treatment, DNA extraction, library construction, sequencing, bioinformatic analysis, and interpretation of data (19). A 1.5ml microcentrifuge tube containing 0.6ml of sample, enzyme, and 1.0g of glass beads (0.5mm) was attached to a horizontal platform on a vortex mixer and agitated vigorously at 2,800–3,200 rpm for 30 min. Then DNA in airway secretions samples was extracted using the TIANamp Micro DNA Kit (DP316, Tiangen Biotech) according to the manufacturer’s instructions. After DNA concentration and purity detection, the libraries were constructed undergoing DNA fragmentation, end-repair, adapter ligation, and PCR amplification. DNA library concentration was measured by Qubit 2.0. An Agilent 2100 test achieved quality control of the DNA libraries. After being pre-quantified by qRT-PCR, quality-qualified libraries were sequenced on the NextSeq™ 550DX platform in SE-75 sequencing type according to the manufacturer’s instructions.

### Data analysis and quality control

Bioinformatics analysis of the mNGS data was performed according to the procedure described in a previous study (20). Raw data (raw reads) were subjected to a quality control process for trimming adapter sequences and removing low-quality tails, reads, and connector sequences using Trimmomatic v0.36 (21). The obtained high-quality and adequate data are called clean reads. Reads mapping to the human genome GRCh37 were removed using the calibration software Bowtie v2.2.6 (22), and the remaining were called unmapped reads (microbial reads). All the microbial reads were deposited in the database under the Sequence Read Archive (SRA) accession number PRJNA932550. Unmapped% refers to the proportion of microbial reads in the clean reads. Duplicated reads introduced in the PCR step were deleted using FASTX-Toolkit, Fulcrum, FastUniq, and CD-HIT-DUP tools (23). Subsequently, Kraken v2.0.9-beta (24) was adopted for the taxonomic classification of microbial reads, with a microbial genome database in NCBI constructed using 51543 genomes of about 27000 species (ftp://ftp.ncbi.nlm.nih.gov/genomes/) (25). The number of reads in the Kraken classification report was further estimated by the Bayesian algorithm named Bracken to produce species-level abundance estimates (26). The estimates of the percentage relative abundance of each species were computed using the reads per kilobase of transcript per million mapped reads (RPKM), a normalization method for mNGS reads, and RPKM was calculated using the formula: gene reads/[the total mapped reads (millions) × genome length (KB)] (27).

### Criteria for defining positive results of mNGS

The mNGS assay was employed for detecting microorganisms, including bacteria, viruses, fungi, and parasites, and a positive result will be judged if it satisfies any of the following criteria described previously (17). 1) The relative abundance of bacteria (excluding *M. tuberculosis* complex) and fungi was greater than 30% at the genera level; 2) Virus detection was considered when the stringent map read number (SMRN) was ≥3. 3) For *M. tuberculosis* complex, at least one number of reads should be aligned to the reference genome at the species or the genus level. However, a positive mNGS finding did not invariably indicate the presence of causative pathogens. Microorganisms detected with mNGS were categorized into colonized, putative, and pathogenic microorganisms. It would be the clinician’s responsibility to determine the putative pathogens and pathogenic microorganisms through comprehensive clinical assessments. In the pathogenic spectrum analysis, the proportion of the pathogenic species, the detection frequency, was calculated with a formulation: the number of samples in which a particular species was detected/the total number of samples.

### Diagnosis of infection and judgment of pathogenic agents

Before and after the lung transplant, the infectious risk and status of the recipients were monitored. The suspicion and diagnosis of infection were based on several clinical symptoms, including body temperature, computed tomography, etiological examination, and immune indicators. In the infected recipients, the putative pathogens and pathogenic microorganisms were judged based on a comprehensive analysis of clinical data, including the number of reads for mNGS, the clinical presentations, radiologic manifestations, conventional detection findings, clinical epidemiology, and the treatment effect of the antibiotic therapy. The putative pathogens or pathogenic microorganisms could be ascertained if the two clinicians approved. Further discussion by senior clinicians is needed in case of a significant disagreement between the first two clinicians. Then, the targeted antibiotic therapy was formulated to fight against infection, and a favorable outcome further confirmed the causative agent. The consistency of mNGS with the causative agents in the infected recipients was evaluated at the species level.

### Statistical analysis

Descriptive statistics were computed for the overall samples and stratified by the positive pathogen detected by mNGS on airway secretions samples. Mean ± standard deviation (SD) or median (interquartile range, IQR) was used for describing the continuous variables. Chi-squared or Fisher’s Exact test was used to compare the two groups’ differences. The significance level was set at 0.05. All statistical analyses were performed using the GraphPad software 8.0.

## Results

### General information of study participants

From October 2018 to June 2022, 40 patients received lung transplant surgery in our hospital, and 38 eligible patients were included for the final analysis. Two recipients were excluded because of death quickly without any microbial culture result. Basic information of these patients was provided in **Supplementary Table 1**. Of all 38 lung transplant recipients, the mean age was 58.13 years (ranges 33-70), including 33 (86.84%) males. The most common primary disease was COPD (19, 50%), followed by interstitial lung disease (18, 47.37%), with the addition of one patient with pneumosilicosis. In terms of the lung transplant types, 23 (60.53%) underwent bilateral transplantation and 15 (39.47%) unilateral transplantation. In the 38 recipients, new-onset infection within one month occurred in 26 (68.42%). These clinical characteristics were recorded and demonstrated in **Table 1**. After lung transplant, the initial antibiotic regimens frequently include Sulbactam/Cefopcrazone and Piperacillin Sodium/Tazobactam Sodium. Immunosuppressant regiments comprise cyclosporin A, tacrolimus, and methylprednisolone.

**Table 1:** Characteristics of the lung transplant recipients.

Characteristics	Values
Lung transplant recipients (n)	38
Median age, y (IQR)	60.5 (52.8-65.3)
Sex (male, %)	33 (86.84%)
Primary indications for lung transplantation, n (%)	
COPD	19 (50%)
Interstitial lung disease	18 (47.37%)
Pneumosilicosis	1 (2.63%)
Types of lung transplantation, n (%)	
Bilateral lung transplantation	23 (60.53%)
Unilateral lung transplantation	15 (39.47%)
Infection status within one month, n (%)	
Infected	26 (68.42%)
Uninfected	12 (31.58%)

COPD, chronic obstructive pulmonary disease.

### Pathogenic spectrum generated by mNGS and traditional microbial culture

**Figure 1:**
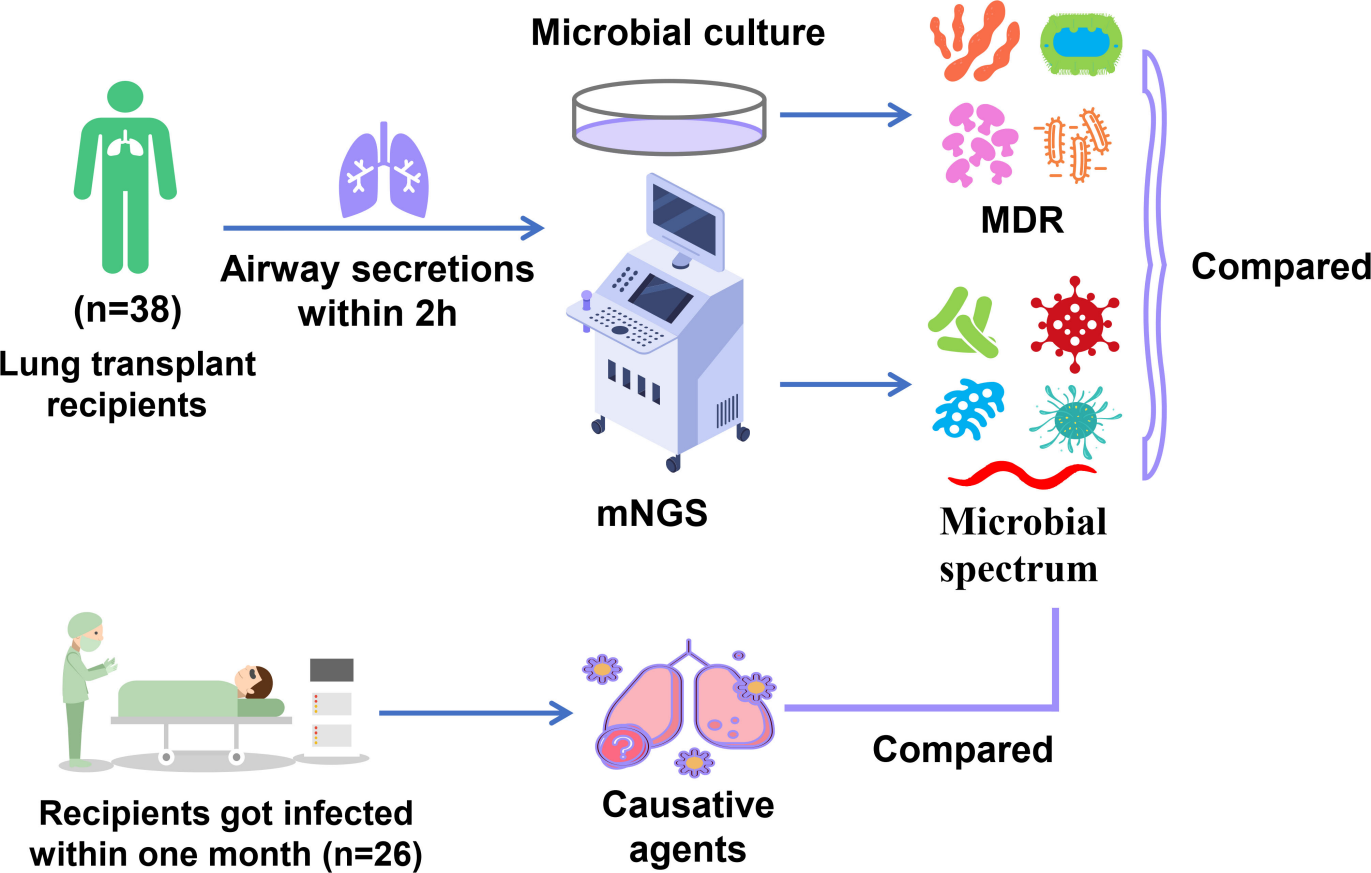
The flowchart of the study design.

38 airway secretions samples from 38 lung transplant recipients were collected within two hours after surgery and simultaneously sent for etiological examination by traditional microbial culture and mNGS. The study design is illustrated in **Figure 1**. The detecting results of the two methods in each patient were provided in **Supplementary Table 1**. This **supplementary material** also included detailed information concerning each sample’s sequencing number of reads (raw reads, clean reads, clean reads/raw reads, unmapped reads, and unmapped %), as well as the putative pathogens in each patient and their relative abundance. It demonstrates that the raw reads range from 4M to 57M, with an average of 20M; most ratios of clean reads to raw reads are above 90%. Unmapped% refers to the proportion of microbial reads in the clean reads, ranging from 0.69% to 79.21%.

**Figure 2:**
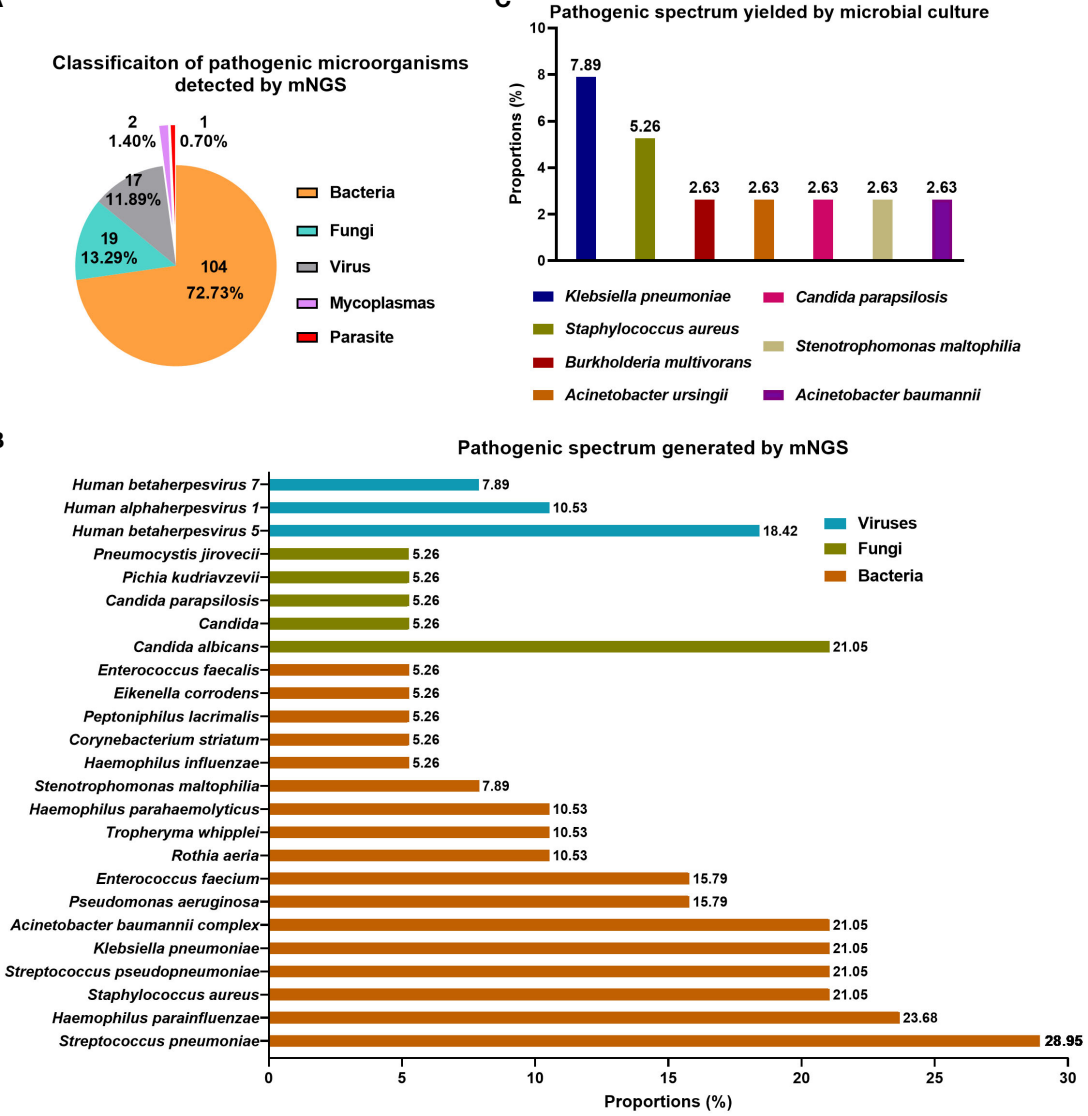
Pathogen spectrum detected by mNGS and traditional culture in airway secretions collected within two hours following lung transplant. **(A)** Classification of pathogenic microorganisms detected by mNGS; **(B)** Pathogenic spectrum detected by mNGS; **(C)** Pathogenic spectrum detected by conventional microbial culture.

143 kinds of pathogenic microorganisms were found in 35 (92.11%, 35/38) airway secretions specimens using mNGS, while the detection rate by microbial culture was 26.31% (10/38) (P<0.0001) (**Table 2**). Statistically, mNGS identified pathogenic microorganisms at the level of species or genus, which were further classified into five types, including bacteria (72.73%), fungi (13.29%), virus (11.89%), mycoplasma (1.4%), and parasites (0.7%) (**Figure 2A**). When analyzed at the species level, *S. pneumoniae* (28.95%) and *H. parainfluenzae* (23.68%) were the top two bacteria, followed by *S. aureus* (21.05%), *S. pseudopneumoniae* (21.05%), *K. pneumoniae* (21.05%), and *A. baumannii complex* (21.05%) (**Figure 2B**). *C. albicans* (21.05%) was the most dominant fungi detected with mNGS. *Human betaherpesvirus 5* (18.42%) was the most prevalent virus. Seven pathogenic microorganisms were detected through the traditional culture method in 10 airway secretions samples. *K. pneumoniae* was detected in three cases (7.89%); *S. aureus* was detected in two samples (5.26%) (**Figure 2C**). The other bacteria include *A. baumannii* and *A. ursingii*, and fungi like *C. parapsilosis* were detected in one sample (2.63%).

**Table 2:** The number of pathogenic microorganisms detected by mNGS and microbial culture in the airway secretions samples collected within two hours from lung transplant recipients.

Samples	mNGS			Microbial culture		Infection status of patients
	Bacteria	Fungi	Viruses	Bacteria	Fungi	
S1	1	0	0	1	0	Infected
S2	13	0	0	1	0	Infected
S3	25	1	1	0	0	Infected
S4	21	1	1	0	0	Infected
S5	1	0	0	0	0	Infected
S6	3	0	2	0	0	Infected
S7	3	0	0	0	0	Infected
S8	13	1	0	1	0	Infected
S9	0	0	0	0	0	Infected
S10	4	0	0	0	0	Infected
S11	12	1	0	1	0	Infected
S12	11	0	0	1	0	Infected
S13	21	3	1	0	0	Infected
S14	16	0	2	0	0	Infected
S15	2	0	0	1	0	Infected
S16	29	1	2			Infected
S17	6	1	2	0	0	Infected
S18	0	1	0	0	0	Infected
S19	22	1	1	0	0	Infected
S20	2	0	0	0	0	Infected
S21	0	0	0	0	0	Infected
S22	22	0	0	0	0	Infected
S23	0	1	0	0	0	Infected
S24	18	0	1	0	0	Infected
S25	2	1	0	0	0	Infected
S26	5	1	2	1	0	Infected
S27	2	1	0	0	0	Uninfected
S28	1	0	0	1	0	Uninfected
S29	1	0	0	1	0	Uninfected
S30	1	1	1	0	1	Uninfected
S31	3	0	0	0	0	Uninfected
S32	0	0	0	0	0	Uninfected
S33	4	0	0	0	0	Uninfected
S34	1	0	1	0	0	Uninfected
S35	2	0	0	0	0	Uninfected
S36	16	0	0	0	0	Uninfected
S37	31	1	0	0	0	Uninfected
S38	16	2	1	0	0	Uninfected

### Time distribution of infection within one month after transplant and the consistency between mNGS-reported pathogens and the causative agents

**Figure 3:**
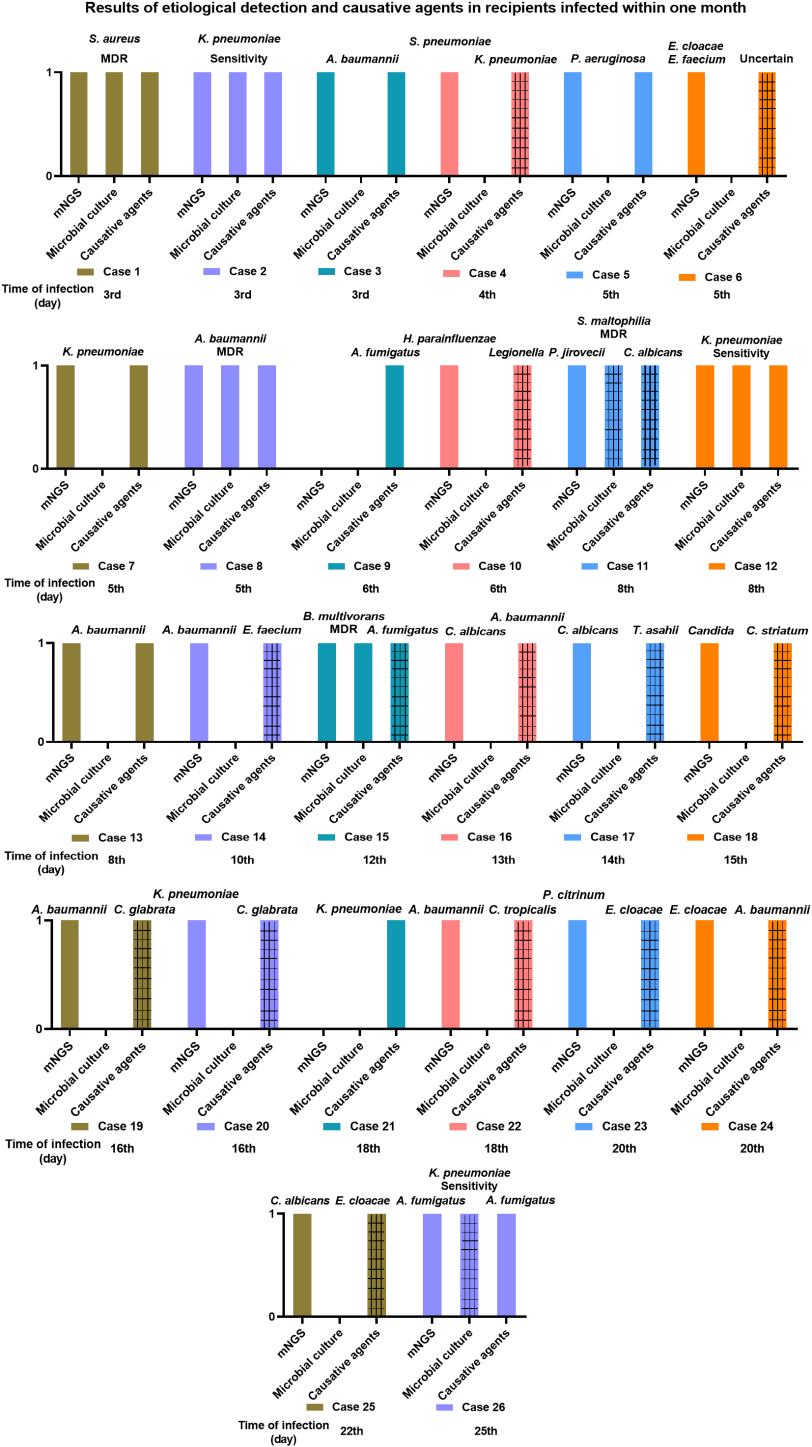
The information of two hours airway secretions mNGS and microbial culture results and the causative agents in recipients infected within one month. The gridlines stand for different pathogenic microorganism when comparing the results of mNGS and microbial culture with the causative agents.

**Figure 3** illustrates the results of etiological identification by mNGS and traditional culture and the information on causative agents in recipients infected within one month. Within one month, 26 (68.42%) of the 38 recipients got infected, and the median time of new-onset infection was 9 days, ranging from 3 to 25 days. Among the 26 infected recipients, 10 (38.46%) got infected within one week following the lung transplant operation, and infection in 7 (26.92%) cases occurred within one to two weeks. The remaining 7 (26.92%) and 2 (7.69%) got infected within two to three weeks and three to four weeks, respectively (**Table 3**). Consequently, infection onset within one week was the highest, and more than half (65.38%) of recipients developed an infection within two weeks. The drug sensitivity of the pathogens was also examined through microbial culture and demonstrated in **Figure 3**. Multiple drug resistance was observed in *S. aureus* (case 1), *A. baumannii* (case 8)*, S. maltophilia* (case 11), and *B. multivorans* (case 15).

**Table 3:** The time distribution of infection within one month in lung recipients.

Onset time of infection	Number of infected cases	Proportions in the infected patients
Within one week	10	38.46%
One-two weeks	7	26.92%
Two-three weeks	7	26.92%
Three-four weeks	2	7.69%

The consistency of two hours airway secretions-mNGS and microbial culture results with the causative agents in the infected recipients was determined at the species level and illustrated in **Figure 3**. In 9 (34.62%, 9/26) infected recipients (patient 1, 2, 3, 5, 7, 8, 12, 13, 26), their causative agents were detected by mNGS in advance (in the airway secretions collected within two hours), who got an infection at the 3rd, 3rd, 3rd, 5th, 5th, 5th, 8th, 8th, and 25th day, respectively, after lung transplant (**Figure 3**). Except for an infection caused by *A. fumigatus* on the 25th day, the median time of infection occurring in the rest 8 recipients was 5 days following the operation. Namely, most of them (6, 66.67%) were infected within one week, 2 (22.22%) cases suffered between one to two weeks, and 1 (11.11%) at three to four weeks (**Table 4**). A decreased trend was observed in consistency, along with the prolonged infection time.

**Table 4:** The consistency of mNGS results in two hours of airway secretions with the causative agents in infected recipients.

Onset time of infection	Number of cases in which mNGS was consistent with the causative agents	Proportions
Within one week	6	66.67%
One-two weeks	2	22.22%
Two-three weeks	0	0%
Three-four weeks	1	11.11%

## Discussion

Our study retrospectively investigated the ultra-early and early etiological characteristic in lung transplant recipients, whose results may provide reference for early antimicrobial strategy in lung transplant recipients. This study completed pathogen identification through the mNGS technology and microbial culture. In general, mNGS performed well in finding diverse microbial species and might serve as an effective supplementary means to traditional etiological detection methods.

In various infectious diseases, the diagnostic accuracy of mNGS is frequently compared with that of conventional detection methods (28). In this study, the traditional culture method served as the control group versus mNGS, whose positive rate for pathogen identification was shallow compared to that of mNGS (26.31% vs. 92.11%). Ju et al. also observed a significantly higher positive rate of mNGS than conventional detection methods (83.4% vs. 55.8%) in airway secretions specimens, with a higher diversity of pathogens simultaneously (17). In our 38 airway secretions samples, mNGS identified 143 kinds of microorganism, ranging from bacteria (72.73%), fungi (13.29%), virus (11.89%), mycoplasma (1.4%), to parasites (0.7%) (**Figure 2A**). The pathogen spectrum revealed that mNGS reported more total amount of pathogen than microbial culture (**Figures 2B, C**). Moreover, mNGS showed absolute superiority in the detection of virus and parasite. Viral infection after lung transplant is common and classified into diseases caused by cytomegalovirus or by other community-acquired respiratory viruses (4, 29). It has been reported that viral pathogens are involved in 25 of 71 infectious episodes in a cohort of lung transplant recipients, with cytomegalovirus-related diseases accounting for 68% of them (8). Without doubt, the conventional diagnosis of parasitic infections in lung transplant recipients is complicated, with clinical suspicion combined with molecular diagnostic methods such as PCR (30). Therefore, the application of mNGS benefits the etiological diagnosis of rare pathogens. To sum up, we claimed that mNGS is superior to the conventional culture in detection rate and in finding more pathogenic microorganisms with a higher diversity, contributing to a wider reference of pathogen screening and the later prophylactic treatment.

Bacterial infections are the most frequent infectious complications. In a Swiss transplant cohort study, 55% of all lung transplant recipients developed infections in the first year, and 63% were bacterial (31). More than half of the pathogens detected in the current study were bacterial microbes, and *S. pneumoniae* (28.95%) and *H. parainfluenzae* (23.68%) were the top two bacteria, followed by *S. aureus* (21.05%), *S. pseudopneumoniae* (21.05%), *K. pneumoniae* (21.05%), and *A. baumannii complex* (21.05%) (**Figure 2B**). They are all the common opportunistic pathogen invading the respiratory tract, and are more likely to invoking infection following lung transplant under immunosuppression (32–34). Thereinto, *S. pneumoniae* and *H. influenzae* are among the main vaccine-preventable bacterial infections in immunocompromised individuals like recipients of solid organ transplants, resulting in a large proportion of hospitalization (34). It has been proven that *K. pneumoniae* is commonly isolated after lung transplantation, and carbapenem-resistant *K. pneumoniae* acquisition is associated with an increased risk of bronchial dehiscence and reduced survival among recipients (33, 35). As reported, fungi are frequently isolated before and after transplantation from respiratory samples, and fungal infections are more common in lung transplant recipients than in most other solid organs (11, 36, 37). In the fungi detected in our samples, *Candida* (34.21%) was the most frequently detected, with *C. albicans* (21.05%) as the predominant species. It led to one infection event in case 11 at 8th day after the operation. *Candida* leads to most fungal extrapulmonary infections in lung transplant recipients, and frequently occurs one-month after the transplant (38). It has been reported that the average period of *Aspergillus*-related infection is 42 days after lung transplantation (12). Our data demonstrated that recipients 9, 15, and 26 were infected by *Aspergillus* on the 6th, 12th, and 25th days after transplant, respectively. In the pathogenic microorganisms identified by mNGS, bacterial pathogens account for more than half (72.73%), with gram-positive and -negative bacteria occupying large proportions. Fungi such as *Candida* are also frequently detected. Therefore, the initial empirical anti-infection regimes covering the bacteria and fungi are reasonable, and the broad spectrum antimicrobial drugs can be substituted by the narrows after the mNGS results produced.

Within one month, 68.42% (26/38) of recipients got infected, and more than half of the infections happened within two weeks. According to **Table 4**, mNGS could predict the causative agents in early infection, especially for the infection onset within one week. Notoriously, donor-derived infections generally manifest during the first few weeks after lung transplant (31). Many deceased donors were more likely to carry pathogens with multiple drug resistance (MDR) or suffered from hospital infections because they stay in the intensive care unit (39, 40). Our **Figure 3** indicated that MDR bacteria were detected in airway secretions samples from 4 cases, and they were *S. aureus* (case 1), *A. baumannii* (case 8), *S. maltophilia* (case 11), and *B. multivorans* (case 15). Bunsow reported that MDR bacteria were isolated from 4.9% (12/243) of donors, including *Enterobacterales*, *S. maltophilia*, *P. aeruginosa*, and *S. aureus *(41). These MDR bacteria should be highly suspected in the cases of infection occurred within 48h or infection worsened after transplant.

In the present studies concerning post-operative infection after lung transplant, many researchers focus on a longer duration, such as three months, one year, even five years (8, 42–44), but early infection within one month has rarely been highlighted. Our study revealed that the median time of new-onset infection was nine days, 38.46% of recipients got infected within one week, and even 65.38% developed infection within two weeks. The high incidence of infection in lung transplant recipients may be associated with the destruction of the mucosal barrier, which was improved with the repair of the mucous membrane (45). Therefore, it is essential to repair the mucosal barrier by removing the tracheal catheters as soon as possible (46). In the infections that occurred shortly after the transplant, the consistency between mNGS results and the etiological agents was high but decreased with the prolonged time interval. That is, the predictive role of mNGS in etiological agents is time-limited, suggesting that continuous pathogenic screening is indispensable for infection prevention (47). With the deepening of research on pathogenic microorganisms affecting lung transplant recipients and advances in pathogen detection technologies, the infection risks are expected to be perceived earlier and specifically intervened to prevent infection and improve their survival rate.

## Data availability statement

The datasets presented in this study can be found in online repositories. The names of the repository/repositories and accession number(s) can be found below: PRJNA932550 (SRA).

## Ethics statement

The studies involving humans were approved by Medical Ethics Committee of Sichuan Provincial People’s Hospital (No. 2021-399). The studies were conducted in accordance with the local legislation and institutional requirements. The participants provided their written informed consent to participate in this study.

## Author contributions

XZ: Conceptualization, Data curation, Writing – original draft, Writing – review & editing. XT: Data curation, Formal Analysis, Investigation, Visualization, Writing – original draft. XY: Data curation, Software, Writing – review & editing. YL: Data curation, Methodology, Writing – review & editing. SL: Data curation, Methodology, Writing – review & editing. TL: Data curation, Methodology, Writing – review & editing. RY: Investigation, Methodology, Writing – review & editing. LP: Investigation, Methodology, Writing – review & editing. GF: Project administration, Software, Supervision, Visualization, Writing – review & editing. XH: Project administration, Software, Supervision, Visualization, Writing – review & editing. YW: Conceptualization, Formal Analysis, Methodology, Writing – review & editing. DC: Conceptualization, Formal Analysis, Methodology, Writing – original draft, Writing – review & editing.
